# Comparative evaluation of platelet rich fibrin matrix (PRFM) membrane and collagen membrane with demineralized freeze-dried bone allograft (DFDBA) in the treatment of mandibular class II furcation defects: A randomized controlled trial

**DOI:** 10.12688/f1000research.131974.1

**Published:** 2023-10-20

**Authors:** Dr. Chitrika Subhadarsanee, Dr. Prasad Dhadse, Dr. Pavan Bajaj, Dr. Mosami Chimote, Dr. Kiran Sethiya, Dr. Komal Bhombe, Dr. Safiya Hassan, Dr. Ranu Oza

**Affiliations:** 1 Assistant Professor, Department of Periodontics, Sharad Pawar Dental College., Datta Meghe Institute of Medical Sciences, Wardha, Maharashtra, 442001, India; 2 Professor and HOD, Department of Periodontics, Datta Meghe Institute of Medical Sciences, Wardha, Maharashtra, 442001, India; 3 Associate Professor, Department of Periodontics, Datta Meghe Institute of Medical Sciences, Wardha, Maharashtra, 442001, India; 4 MDS, Department of Periodontics, Datta Meghe Institute of Medical Sciences, Wardha, Maharashtra, 442001, India; 5 Post graduate student, Department of Periodontics, Datta Meghe Institute of Medical Sciences, Wardha, Maharashtra, 442001, India

**Keywords:** Class II Furcation, Guided tissue regeneration, PRFM, DFDBA

## Abstract

**Aim-** The aim of the study was to compare the effectiveness of platelet rich fibrin matrix (PRFM) membrane with collagen membrane (Colo Gide) in combination with demineralized freeze-dried bone allograft (DFDBA) in the treatment of mandibular Class II furcation defects.

**Methods-** This randomized, parallel designed, controlled, clinical investigation was conducted in 24 subjects (15 male and 9 female) having Class II furcation defects either buccally or lingually. The test group was treated with DFDBA and PRFM membrane while the control group was treated with DFDBA and collagen membrane. The clinical measurements such as plaque index (PI), papillary bleeding index (PBI), pocket probing depth (PPD), relative attachment level (R-CAL) and relative gingival marginal level (R-GML) were measured at baseline and six months. Radiographic parameters, such as vertical defect depth (VDD), horizontal defect depth (HDD) and defect width (DW) were measured using cone beam computed tomography taken at baseline, three and six months. Student’s paired t-test was utilized to analyse data from the day of surgery to six months. A comparison of both groups at baseline and six months was achieved by student’s unpaired t-test.

**Result-** 10 sites in test group (83.33%) showed the advancement from class II to class I compared to eight sites in control (66.66%). Remaining defects in test group n=2 (16.66%) and control group n=4 (33.33%) showed marked reduction in horizontal defect depth compared to baseline. No complete closure of the defect was seen in either group.

**Conclusion-** When treating class II furcation defects, the use of PRFM membrane combined with DFDBA seems to be advantages with regards to collagen membrane. The presented set up seems feasible with regards to randomization, acceptance, retention and achievement of satisfactory outcomes.

## Introduction

Invasion of furcation areas in multirooted teeth presenting bifurcation and trifurcation always poses continuous challenge to the clinician owing to complex furcal anatomy, difficulty to perform adequate personal oral hygiene and effective periodontal instrumentation. ^1^ Thus, the prognosis of a tooth with a furcation involvement (FI) is always bleak. ^2^^,^^3^ Depending on the severity of the furcation defect, a variety of therapeutic options such as odontoplasty, ^4^ furcationplasty, ^5^ tunnelling ^6^ and guided tissue regeneration (GTR) employing different bone grafts, ^7^ barrier membranes ^8^ alone or in combination are available for treatment. Recently, platelet concentrates containing growth factors ^9^ have also been investigated. ^10^ GTR includes the elimination of both epithelial and connective-tissue cells of the gingiva from denuded root surface, allowing periodontal ligament (PDL) or alveolar bone cells to repopulate the wound region. ^11^ When compared to monotherapeutic algorithms, the use of a combination therapeutic strategy ( *i.e.*, bone-replacement graft, barrier with or without biologics) provides an added benefit and excellent predictability for regeneration of periodontium in furcation defects. ^6^^,^^9^^,^^12^^,^^13^ At this time, there is no specific regenerative material that is regarded the gold standard for the management of furcation defects. ^14^ Clinicians are still investigating for a “off-the-shelf” material that can substitute and/or improve grafting procedure while also providing improved, additional reliable clinical outcomes than existing bone scaffolds and matrices. ^15^ In recent years, growth factors (GFs) are in the limelight in the craniomaxillofacial and periodontal sectors. ^16^^,^^17^

Decalcified freeze-dried bone allograft (DFDBA) works as a source of osteo-inductive substances while providing an osteoconductive surface. ^18^ Bone morphogenic proteins (BMPs) such as BMP 2, 4, and 7 are found in DFDBA and serve to trigger osteo-induction. ^19^ Thus, allograft proteins manufactured commercially have the ability to alter cell activity *in vivo.* ^20^ Clinical research investigations have shown that using DFDBA in human intraosseous ^21^^,^^22^ lesions improved clinical attachment and bone level, with histological evidence of new attachment formation. ^23^^,^^24^

GTR effectively prevents tissue and bone degradation while also stimulating the development of new tissue and bone. ^25^ In order to cover the region where the regeneration process will take place, a physical barrier (membrane) with the correct shape and position is required. GTR was originally reported to be effective in regenerating damaged periodontal tissues in class II furcation involvement. ^26^ Their biggest drawback is their lack of stiffness, which limits their ability to create space and necessitates the use of a scaffold. Collagen membranes, on the other hand, can be employed alone for alveolar bone defects such as bone dehiscence and fenestration defects that do not require further fixation and stability. ^27^ Furthermore, because they degrade quickly, they may not be able to satisfy the time requirements for good tissue growth.

A new generation of platelet-rich fibrin matrix (PRFM) is a concentrate of platelets that require no biological components for its preparation (bovine thrombin). ^28^ PRFM structure looks like natural fibrin and promotes cell motility, proliferation, and cycle creation. ^29^ On the first day, levels of platelet-derived growth factor (PDGF), vascular endothelial growth factor (VEGF), basic fibroblast growth factor (bFGF) and transforming growth factor beta (TGF-β) are increased, then gradually tend to decrease the next day. ^30^^,^^31^ PRFM also exhibits fibrin properties, such as a denser and more flexible macroscopic structure and a more natural platelet distribution. ^32^^,^^33^ Because of these variables, this preparation behaves more like a natural clot, releasing low concentrations of growth factors over a longer period of time. ^30^^,^^34^ To the best of our knowledge the use of PRFM membrane in periodontal regeneration is scarce and limited.

Three dimensional (3D) imaging, such as cone beam computed tomography (CBCT), can offer details regarding faults that aren’t visible in two dimensional 2D photos. ^35^ It was employed to compensate for the limitations of two dimensional (2D) scanners and to clearly show the horizontal component of the furcation. It can be used to evaluate treatment outcomes, particularly to check healing following grafting or regeneration. This imaging technique may also be used to measure the gingival tissue and the dimensions of the dentogingival unit. ^36^^,^^37^ CBCT is used to assess and plan treatment for molars with furcation involvement by revealing marginal bone contouring, intra-bony and furcation defects. ^38^

Therefore, the following investigation has been undertaken to compare the effectiveness of PRFM membrane with that of collagen membrane (Colo Gide) in combination with DFDBA in the treatment of defects with Class II furcation, both clinically and radiographically.

## Methods

The current randomized, parallel designed, controlled, clinical study was conducted in 24 subjects (12 in each group) as determined by sample size calculation using two means with equal variance (15 male and 9 female), with moderate to advanced chronic periodontitis having Class II furcation defects either buccally or lingually with age range of 25 to 50 years with a mean age of 40.5 were selected from Department of Periodontics. Subjects were recruited on an outpatient basis and separated into groups by computer generated random number table. This study design protocol was ethically passed by the “Institutional Ethical Committee” (DMIMS (DU)IEC/2017-18/6730). All participants were informed verbally and written informed consent was obtained for the surgical procedure. The CONSORT Checklist and guidelines were followed for this study. ^39^^,^^40^

Healthy subjects without any systemic illness with horizontal probing depth (HDD) and vertical furcation probing depth (VDD) of ≥3 mm, proximal bone height coronal to the inter-radicular bone level and radiographic evidence of furcation defects in the molars (buccal, lingual, mesiobuccal or distobuccal) were included. To match all the characteristics of included subjects at baseline the age group of the subjects were in the range of 25 to 50 years.

Subjects with poor oral hygiene following “etiotropic/phase I periodontal therapy” and exhibiting plaque scores greater than or equal to 1, mobile teeth, any systemic diseases, suspected or known allergies to drugs or study materials, use of tobacco in any form, immunocompromised subjects, alcoholics, lactating or pregnant women, and subjects with compromised immune systems were excluded.

Prior to surgery, after initial treatment (scaling and root planing), the selected defects were randomly assigned to the test and control groups, each of which had 12 defects, using a computer-generated random number table (Calculator Soup ^®^ Online Calculators). During the allocation system, both the participants and the examiner were blinded. In contrast to the control group, which received treatment from collagen membrane (Colo Gide) and DFDBA (n=12), the test group was handled by PRFM and DFDBA. The assessment of outcomes involved re-evaluating patients after three and six months of treatment.

**Figure 1.**
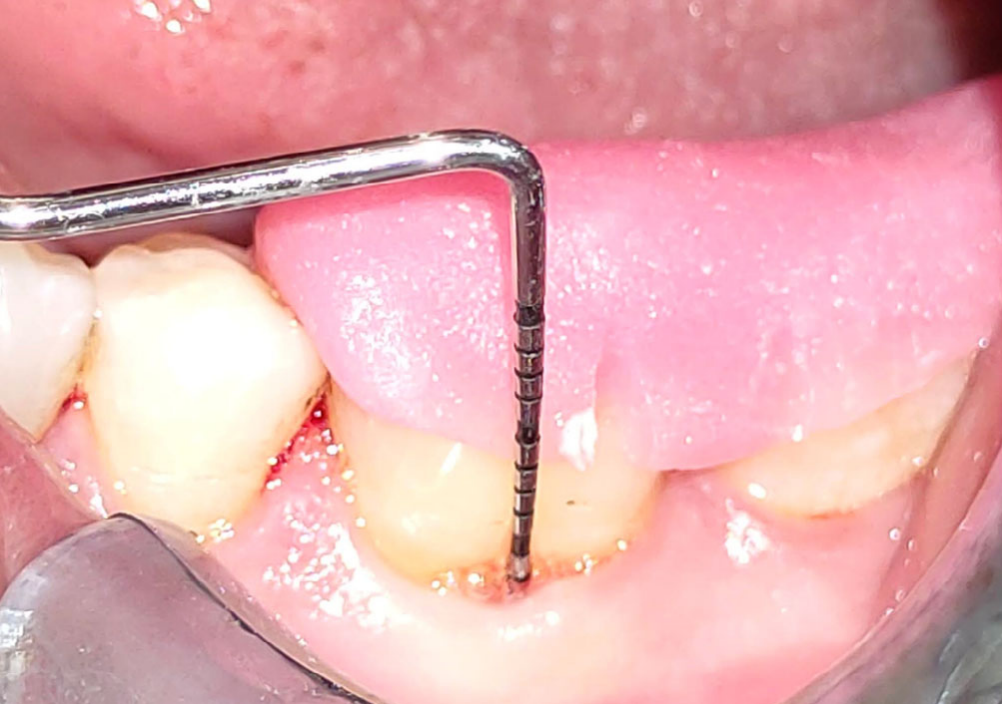
Measurement of vertical probing depth (VPD) using UNC-15 probe.

To standardise the probe location and angulations to assess the horizontal and vertical probing depth before the procedure and after six months, specially designed occlusal acrylic stents were created ( Figure 1). For the purpose of creating a cast model of both jaws, alginate impressions were made. On the cast model, an acrylic stent was made. The treated tooth’s occlusal surface and at least one tooth’s distal and mesial surfaces were all covered by the occlusal stent. A reference point was established for the placement of the periodontal probe in the deepest part of the affected tooth. The stent’s apical border was used as a fixed reference point. (Fixed reference point was made at baseline to replicate the same position at the following visit, thus avoiding the variability in measurement).

Plaque index (PI) by Silness and Loe, ^41^ papillary bleeding index (PBI) by Saxer and Mühlemann, ^42^ probing pocket depth (PPD), relative attachment level (R-CAL), and relative gingival marginal level (R-GML) were the clinical parameters that were recorded. On the first day of therapy and three and six months following the procedure, all clinical signs were assessed. The radiographic bone fill was the study’s primary outcome, while improvements in relative attachment level (R-CAL) and probing pocket depth (PPD) were its secondary outcomes.

PI by Silness and Loe was graded from score 0-3 according to the accumulation of microbial plaque with respect to free gingival margin. ^41^

PBI by Saxer and Mühlemann was graded from score 0-4 according to the intensity of any bleeding with UNC 15 probe which was inserted into the gingival sulcus at the base of the papilla on the mesial aspect, and then moved coronally to the papilla tip. This is repeated on the distal aspect of the papilla. ^42^

A UNC-15 (University of North Carolina, Hu-Friedy, Chicago, USA) probe was measured from the inferior border of the acrylic stent referred as relative gingival marginal level (R-GML). At three locations on each furcation surface—distal, mesial line angle, and midbuccal or midlingual surface—the relative attachment level (R-CAL) was measured using the distance between the base of the pocket and the inferior border of the stent using the UNC-15 probe. The UNC-15 probe is used to measure PPD from the gingival margin to the base of the pocket. The vertical probing depth (VPD), measured from the pocket base to the gingival margin, was estimated ( Figure 1). ^43^^,^^44^ Horizontal probing depth (HPD) of furcation was measured by a curved color-coded furcation probe (GDC Double End Probes Nabers Color Coded # 6 (Pq2n)) with 0-3, 3-6, 6-9 and 9-12 mm markings. The width of keratinized gingiva (WKG) was calculated from apical most point of mucogingival junction to the crest of gingival margin using a UNC-15 probe. All the probing measurements were noted at baseline (pre-operation), three and six months post-operatively.

The caliper included with in the cone beam computed tomography (CBCT) (Planmeca Promax 3D G-XR-109125, software- Romexis viewer) was used to evaluate radiographic parameters. The vertical defect depth (VDD) was measured in sagittal view from the fornix to the base of the defect, and the horizontal defect depth (HDD) was measured in axial section from a tangent line linking the greatest convexities of the mesial and distal roots to the deepest area of the defect. In sagittal view, the defect width (DW) was assessed.

**Figure 2.**
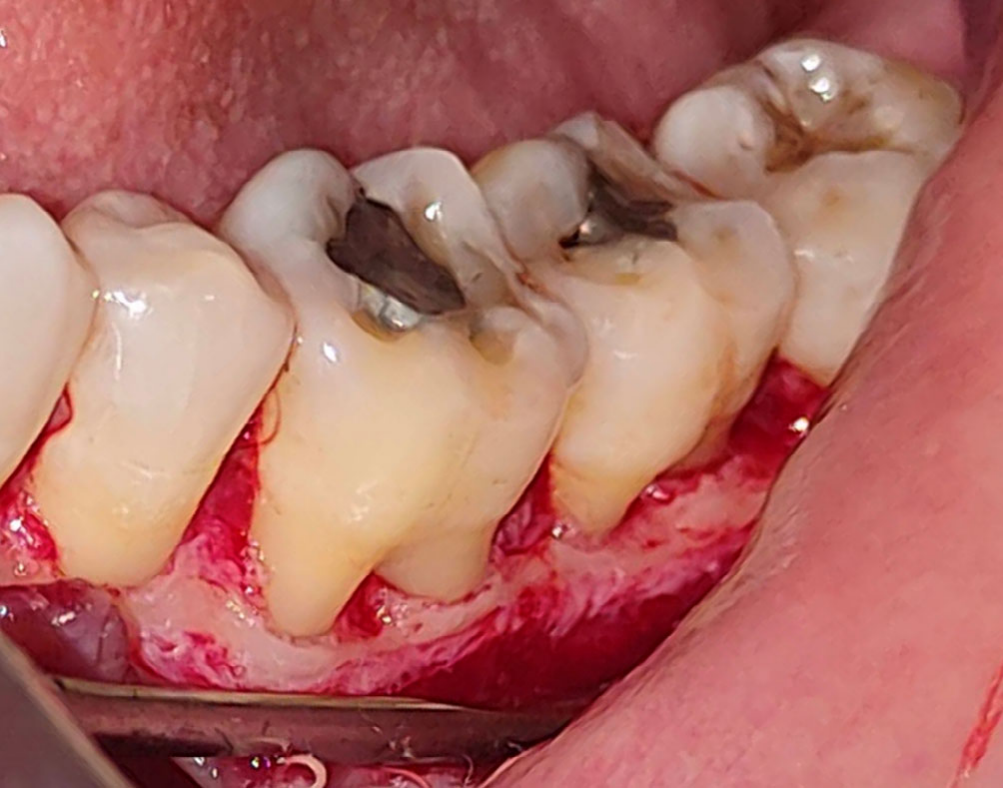
Full thickness flap reflection.

**Figure 3.**
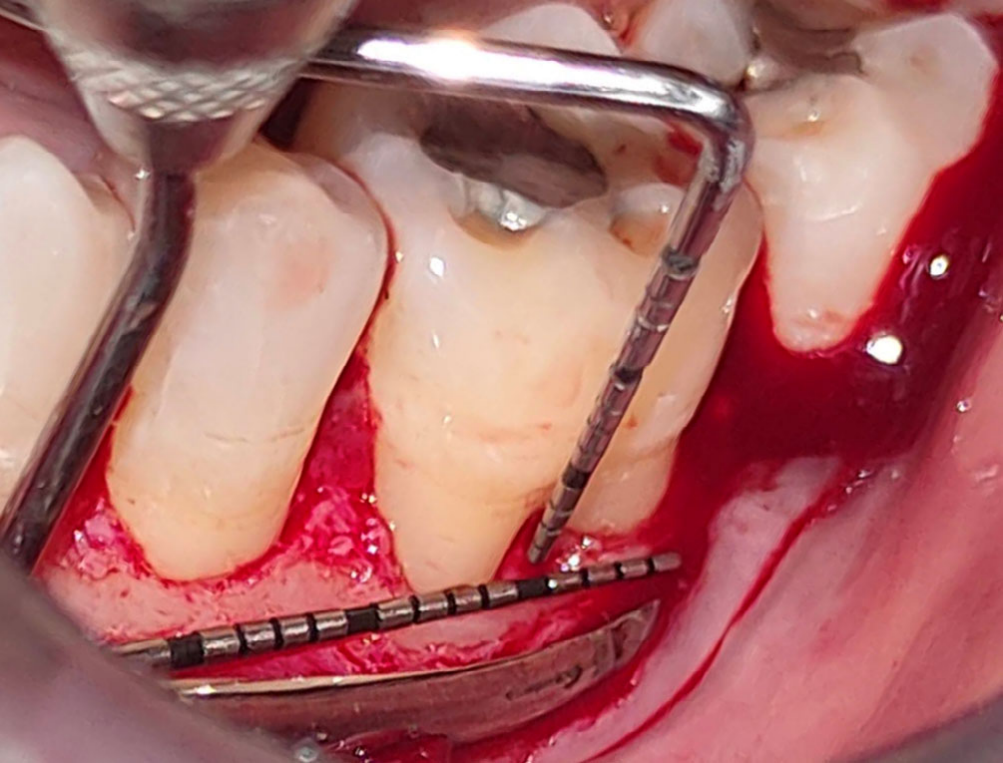
Intrasurgical vertical defect depth measurement.

All study participants underwent a pre procedural mouth rinse with a 0.2% chlorhexidine gluconate solution for one minute. The surgical procedure was conducted while maintaining asepsis. The area was anaesthetized by nerve block and infiltration using 2% Xylocaine with a 1:80,000 epinephrine concentration (Ligno-Ad local anaesthetic, Proxim Remedies, India). Surgical blade no. 15 was used to create intra-crevicular incisions on the lingual or buccal surfaces of the affected tooth. The interdental papillae were secured by the interproximal incisions in order to achieve primary wound closure. The flap also covered the tooth’s mesial and distal portions. With a periosteal elevator (24 G Hu-Friedy, USA) on the affected site, a mucoperiosteal flap was raised to reveal the underlying defect margin ( Figure 2). ^43^^,^^44^ With the aid of hand instruments like universal curettes (GDC Universal Curettes 4r/4l Posterior SC4R/4L - 3), an ultrasonic instrument (Woodpecker HW-3H), and furcation curettes (GDC Furcation Curette Quetin - Buccal/lingual # 6 (SQBL2 # 6), the denuded root surface with the furcation dome was debrided. The root surfaces and furcation flaws were planed until a smooth, firm consistency was reached. After achieving hemostasis and irrigation with physiological saline solution, intraoperative measurements of both HDD and VDD at the furcation site were taken ( Figure 3). ^43^^,^^44^ 1) HDD: The furcation defect’s deepest region was measured horizontally with a UNC 15 probe, and a second UNC 15 probe was put at the prominence of the root surface to bridge the first probe as a reference. 2) Using the furcation fornix as a fixed reference point, the vertical furcation defect measurement (VDD) was taken. The final patient’s eligibility for the trial was confirmed if the furcation defect depth was 3 mm both vertically and horizontally. The trial does not exclude any patients. Following the intraoperative measurements, the sites were then assigned to the test or control group using a computer-generated random number (Research Randomizer, RRID:SCR_008563).

### Procedure for control group

**Figure 4.**
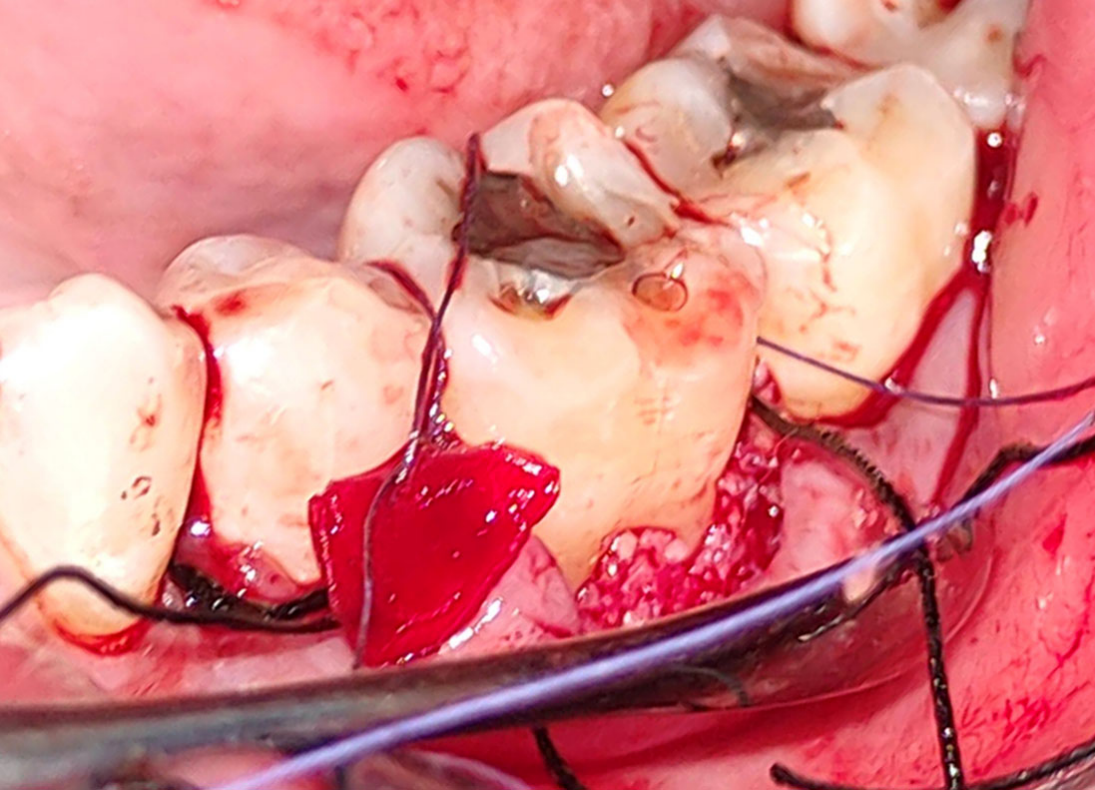
Demineralized freeze-dried bone allograft (DFDBA) placed in furcation defect.

**Figure 5.**
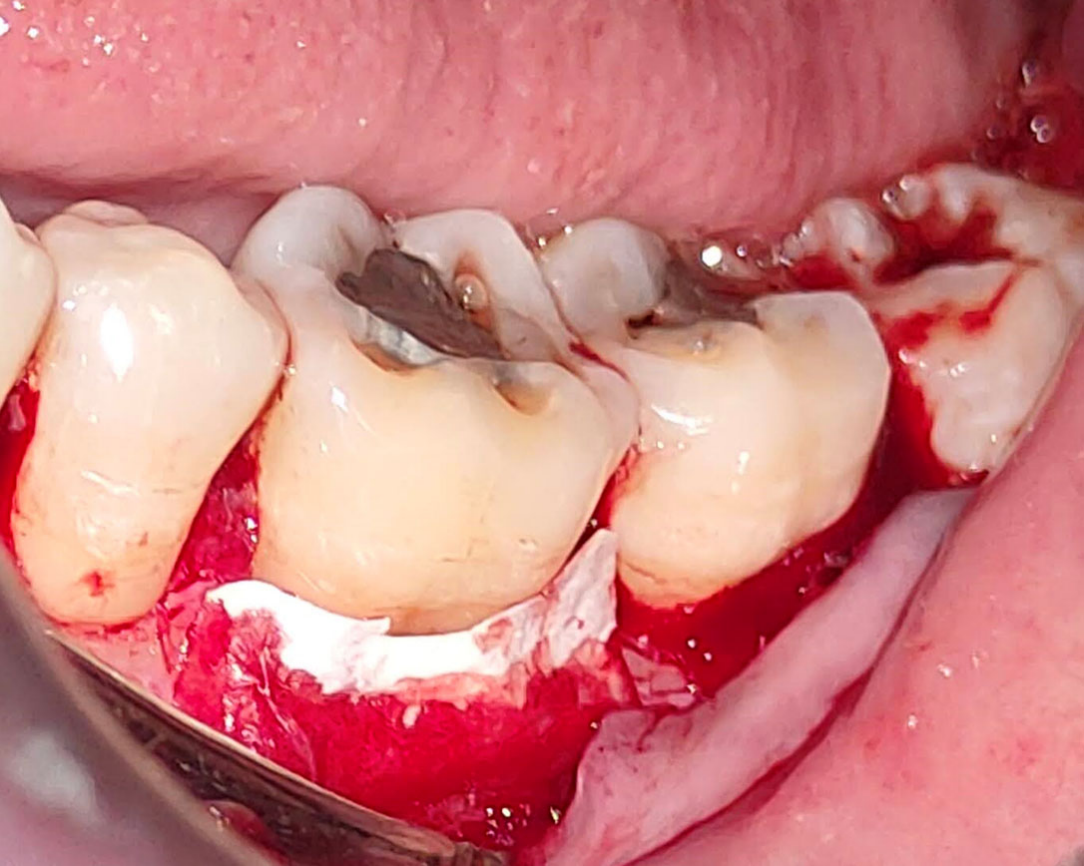
Guided tissue regeneration (GTR) membrane placed in the furcation defect.

**Figure 6.**
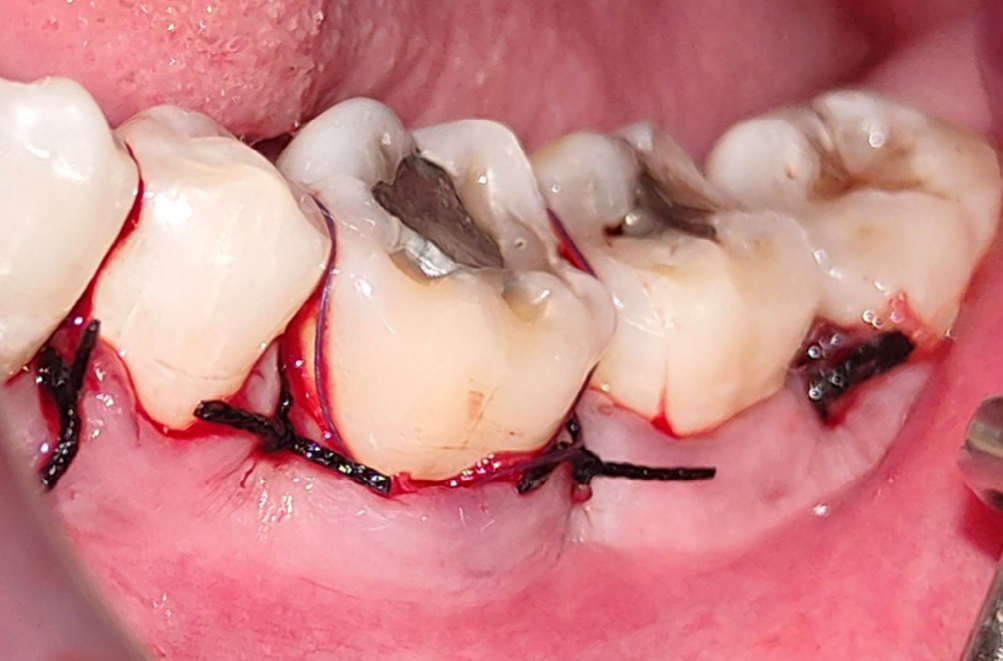
Flap sutured.

Complete isolation and hemostasis of site was obtained using gauge piece manual pressure application with one finger (Hospital Cotton Woven Fabric, Check/stripes, White) and suction tube (PDD suction tip). After complete debridement of furcation defect, DFDBA was condensed ( Figure 4) ^43^^,^^44^ and covered by Cologide™ bioabsorbable GTR membrane ( Figure 5). ^44^ The membrane will be stabilized and then the flap was sutured ( Figure 6). ^43^^,^^44^ Periodontal pack was given.

### Procedure for test group (making of PRFM membrane)

In test sites PRFM membrane ( Figure 5) ^43^ along with DFDBA ( Figure 4) ^43^^,^^44^ was used. A 10 millilitre sample of blood was collected from antecubital vein and transferred in Merisis PRFM test tubes (Meresis, Laboratory, Bengaluru, Karnataka, India). Then in single time centrifugation (Remi R-8C 16×15 ml Laboratory Centrifuge with Angle Rotor Head) technique PRFM clot was made with 3000 rpm for 10 min. The upper layer of clot was removed and with the help of PRF box PRFM membrane was obtained and placed in the furcation defect.

### Post-operative care

Following the procedure, a non-steroidal anti-inflammatory medication (Ibugesic Plus ^®^, Cipla Pharmaceuticals, India) containing a combination of Ibuprofen 400 mg and Paracetamol 325 mg, as well as the antibiotic Amoxicillin 500 mg three times daily, were prescribed for five days. For 4-6 weeks, it was recommended to all subjects to gargle with 0.2% chlorhexidine gluconate twice daily for one minute. Patients were advised to protect the pack from any harm. After one week, the periodontal dressing and sutures were removed, and recovery was shown. Subjects were instructed to use a gentle toothbrush and cotton pellets to clean the surgical site in an apico-coronal manner. One, three, and six months following treatment, the individuals were recalled.

### Statistical analysis

All clinical parameters, including PPD, HPD, CAL, and GML, were estimated using the mean and standard deviation (Mean SD) results. Data from the day of surgery to six months were analysed using a paired t-test with students as the subjects. Student’s unpaired t-test allowed for a comparison of the two groups at both their baseline and six-month points in time. The paired t-test used by the students was used to compare the PI and PBI at baseline and after six months. If the probability value (p) is less than 0.05, the difference was ruled non-significant; if it is greater than 0.05, it was considered significant. To determine differences among each group, the Wilcoxon test or paired student’s t-test was used. All hard and soft tissue variables were compared between the test and control groups using the Mann-Whitney test or an independent student’s t-test. In all phases of assessment, a p value <0.05 was considered significant. All data were assessed using SPSS 11.0 (SPSS inc, 2003) (RRID:SCR_002865) software.

## Results

A total of 24 class II mandibular furcation defects (First molars n=17; second molars n=7) involving either buccal (n=22) or lingual (n=2) surfaces in 18 subjects were treated in this study. Patients involved in the study with age ranged between 25 to 50 years (Male:Female - 15:9). All the clinical and radiographic parameters are provided in the masterdata. ^45^

During the time slot of six months, healing was uneventful, all patients reported for post-surgical evaluation therefore the study did not exclude any of the sites. All selected patients reported for the therapy and follow up after three and six months. All the participants reported satisfaction with the treatment given to them.

Comparison between baseline PI and PBI score to six-month follow-up revealed a significant decline in both groups. (p<0.05). Low PI score (<1) in both the groups indicated that good plaque control was maintained throughout the study period indicating that the gingival inflammation was reduced and the tissues remained healthy ^46^ ( Table 1).

**Table 1.** Comparison of the mean plaque index (PI) and papillary bleeding index (PBI) scores between baseline and six months follow-up in Demineralized freeze-dried bone allograft + platelet rich fibrin matrix membrane (test) and Demineralized freeze-dried bone allograft + Colo Gide (control) groups (Mean±SD; in mm).

Parameters	Group	Baseline	6 months	Difference	*p* value
**Plaque index (PI)**	**Test**	0.77±0.07	0.54±0.05	0.23±0.08	<0.001, S* ( *p*<0.05)
	**Control**	0.75±0.06	0.51±0.05	0.23±0.04	<0.001, S* ( *p*<0.05)
**Papillary bleeding index (PBI)**	**Test**	0.7±0.08	0.47±0.06	0.22±0.10	<0.001, S* ( *p*<0.05)
	**Control**	0.67±0.06	0.43±0.04	0.24±0.07	<0.001, S* ( *p*<0.05)

**NS=Non-significant** ( *p*>0.05).

*P* value is calculated based on the data and less than 0.05 is statistically significant.

All the investigated parameters in both groups at baseline were observed to be statistically non-significant (p>0.05), indicating same starting point for both procedures. Clinical parameters VPD, R-CAL, HPD and RGML revealed a significant reduction (p<0.05) after six months compared to baseline in both groups ( Tables 2 and 3).

**Table 2.** Comparison of the clinical parameters between baseline, three months and six months follow-up in test group (Mean±SD; in mm).

Parameters	Baseline	3 months	Difference	*p* value	6 months	Difference	*p* value
**Vertical probing depth** ( **VPD)**	4±0.85	2.5±0.52	1.5±0.52	**<0.001**	**1.5**±0.52	**2.5**±0.52	**<0.001**
**Relative attachment level** ( **R-CAL)**	13.75±1.86	12.41±1.56	1.33±0.88	**<0.001**	**11**±2	**2.75**±0.62	**<0.001**
**Horizontal probing depth** ( **HPD)**	4.08±1.44	2.75 ±0.96	1.33±0.49	**<0.001**	**1.58**±0.52	**2.5**±0.79	**<0.001**
**Relative gingival marginal level** ( **RGML)**	9.41±1.08	9.5±1.08	0.08 ±0.51	**0.58**	**9.16**±1.40	**0.25**±0.62	**0.19**

*P* value is calculated based on the data and less than 0.05 is statistically significant.

**Table 3.** Comparison of the clinical parameters between baseline, three months and six months follow-up in control group (Mean±SD; in mm).

Parameters	Baseline	3 months	Difference	*p* value	6 months	Difference	*p* value
**Vertical probing depth** ( **VPD)**	3.91±0.79	2.75±0.45	1.16±0.57	**<0.001**	**1.58**±0.51	**2.33**±0.65	**<0.001**
**Relative attachment level** ( **R-CAL)**	13.5±2.02	12.58±2.35	0.91±0.99	**0.008**	**11.16**±2.40	**2.33**±0.65	**<0.001**
**Horizontal probing depth** ( **HPD)**	4.33±1.23	3±0.85	1.33±0.49	**<0.001**	**1.91**±0.90	**2.41**±0.66	**<0.001**
**Relative gingival marginal level** ( **RGML)**	9.75±2.26	10±2.41	0.25 ±0.62	**0.19**	**9.75**±2.26	**0**	**1**

*P* value is calculated based on the data and less than 0.05 is statistically significant.

Comparison between mean VPD reduction in test (2.5±0.52 mm) and control group (2.33±0.65 mm) at six months indicated non-significant (p-0.43) in both group by 0.16±0.71 mm. Comparison of mean CAL gain among groups at six months indicated no statistically significant difference (0.41±1.08 mm). Comparison of mean CAL gain among groups at three months indicated no statistically significant difference (0.41±1.56 mm) ( Table 5). The mean reduction of HPD for test group (0.17±0.62 mm) when compared with control group (2.41±0.66 mm) at six months, found to be statistical non-significance difference (p>0.50). The mean gain of gingival marginal level for test group (0.25±0.62 mm) when compared with control group (0 mm) later at six months, considered be statistical non-significance (p>0.50) ( Table 6).

**Table 4.** Hard tissue measurements of the sites using Cone beam computed tomography (CBCT) for test & control group (Mean±SD; in mm).

	Test group				Control group			
Parameters	Baseline	6 Months	Difference	*p* value	Baseline	6 Months	Difference	*p* value
**Horizontal defect depth (HDD)**	3.31 **±0.97**	1.77 **±0.38**	1.56 **±0.85**	**<0.001**	3.73 **±0.54**	1.94 **±0.38**	1.79 **±0.48**	**<0.001**
**Vertical defect depth (fornix to base of defect) (VDD)**	1.43 **±0.54**	0.97 **±0.57**	0.45 **±0.53**	0.01	1.51 **±0.45**	0.81 **±0.21**	0.7 **±0.27**	**<0.001**
**Defect width (DW)**	1.93 **±0.43**	1.12 **±0.46**	0.81 **±0.40**	**<0.001**	2.11 **±0.32**	1.29 **±0.28**	0.82 **±0.16**	**<0.001**

*P* value is calculated based on the data and less than 0.05 is statistically significant.

**Table 5.** Comparison of the clinical parameters between test and control groups at 3 months follow-up. (Mean±SD; in mm).

Parameters	Test group	Control group	Difference	*p* value
**Vertical probing depth reduction**	1.5±0.52	1.16±0.57	0.33±0.65	**0.10**
**Clinical attachment level gain**	1.33±0.88	0.91±0.99	0.41±1.56	**0.37**
**Horizontal probing depth reduction**	1.33±0.49	1.33±0.49	0±0.73	**1**
**Increased gingival marginal level**	0.08 ±0.51	0.25 ±0.62	0.16±0.93	**0.55**

**S=Significant** ( *p*<0.05); **NS=Non-significant** ( *p*>0.05).

*P* value is calculated based on the data and less than 0.05 is statistically significant.

**Table 6.** Comparison of the clinical parameters between test and control groups at 6 months follow-up. (Mean±SD; in mm).

Parameters	Test group	Control group	Difference	*p* value
**Vertical probing depth reduction**	**2.5**±0.52	**2.33**±0.65	0.16±0.71	**0.43**
**Clinical attachment level gain**	**2.75**±0.62	**2.33**±0.65	0.41±1.08	**0.20**
**Horizontal probing depth reduction**	**2.58**±0.79	**2.41**±0.66	0.17±0.62	**0.75**
**Increased gingival marginal level**	**0.25**±0.62	**0**	0.25±0.62	**0.19**

**S=Significant** ( *p*<0.05); **NS=Non-significant** ( *p*>0.05)

*P* value is calculated based on the data and less than 0.05 is statistically significant.

Radiographic parameters HDD & DW showed statistical significantly (p<0.001) result at six months compared to baseline in both the groups where VDD signifies non-significant (P- 0.01) result ( Table 4). This can be appreciated on radiographic images which can be found in the extended data. ^47^^,^^48^

The mean reduction of HDD for test group (1.56±0.85 mm) in comparison with control group (1.79±0.48 mm) at 6 months, showed statistical non-significance difference (p>0.40). Comparison of mean VDD gain between both groups at 6 months indicated non-significant (p-0.26) difference (0.25±0.70 mm). The mean gain in DW for the test group (0.81±0.40 mm) when compared with control group (0.82±0.16 mm) at the end of study considered statistically non-significance (p-0.93) ( Table 7).

**Table 7.** Comparison of the radiographic parameters between test and control groups at 6 months follow-up. (Mean±SD; in mm).

Parameters	Test group	Control group	Difference	*p* value
**Horizontal defect depth (HDD)**	1.56 **±0.85**	1.79 **±0.48**	0.23±0.92	**0.40**
**Vertical defect depth (fornix to base of defect) (VDD)**	0.45 **±0.53**	0.7 **±0.27**	0.25±0.70	**0.26**
**Defect width (DW)**	0.81 **±0.40**	0.82 **±0.16**	0.01±0.42	**0.93**

**S=Significant** ( *p*<0.05); **NS=Non-Significant** ( *p*>0.05).

*P* value is calculated based on the data and less than 0.05 is statistically significant.

A total of 10 sites in test group (83.33%) showed the advancement from class II to class I compared to eight sites in control (66.66%). Remaining defects in test group n=2 (16.66%) and control group n=4 (33.33%) showed marked reduction in horizontal defect depth compared to baseline. No closure of the defect was seen completely.

## Discussion

The success of furcation treatment is based on eradication of both horizontal and vertical defect components, as well as the improvement of clinical parameters. ^46^ Various therapeutic techniques were created and tested in order to accomplish the anticipated result in the management of class-II mandibular FIs. AAP regeneration workshop ^13^ stated the use of a combination treatment strategy to regenerate the periodontium that appears to have an advantage over monotherapeutic techniques. One of the approaches is GTR that has been established as a successful therapeutic alternative with high predictability for the treatment of different furcation type defects, particularly Class II defects. The current study used a bone graft DFDBA that promotes host’s undifferentiated mesenchymal cells to develop into osteoblasts, resulting in the production of new bone. ^49^

The mean PPD reduction (0.16±0.71 mm; p=0.43) in our study is supported by evidence, which shows PPD decrease by employing DFDBA with and without PRF in the management of class II furcation defects and showed statistical significant PI score (p<0.001) at six months compared to baseline. ^17^^,^^50^ In this study, the test group had a gain RGML (0.25±0.62) than the control group (0). Sharma *et al.* ^51^ found that the PRF group (0.344±0.086) had similar GML alterations to the OFD group (0.756±0.115) in their study.

Another criterion is clinical attachment level gain that may be used to justify good clinical periodontal regeneration following periodontal treatment. In the present study, both groups showed no statistically significant difference for mean CAL gain- 0.41±1.08; p=0.20. Observations made in the present study with regards to clinical attachment gain are comparable with results stated in a study conducted by Basireddy *et al.* (2018) ^17^ and Mehta *et al.* (2018) ^50^ where statistical non-significant difference found in RHCAL (p= 0.055) with combination therapy. The use of PRFM and collagen membrane along with bone graft has led to a demonstrable CAL gain in furcation defects, thus indicating a regenerative potential of both the membranes. In the present study, gain in HDD, VDD and DW using CBCT in both groups substantiated the bone filling potential of both PRFM membrane and collagen membrane with DFDBA.

Third-generation platelet concentrates include autologous growth factor concentrates as well as PRFM. ^29^ The structural alterations in the platelet concentrate’s fibrin gel are caused by a variety of factors, including thrombin- fibrinogen concentration ratios and protein and ion concentrations, including calcium. It has been observed that not only platelets, but also leukocytes are concentrated as studied in the intricate 3-D design. ^52^ Due to the strong fibrin matrix, PRFM resorbs gradually, allowing for a prolonged release of platelet and leukocyte-derived GFs into the wound region from seven to 23 days. ^31^^,^^34^ It speeds up healing, is inexpensive, and may be used on major bone deformities. Microscopically, it is formed of amorphous fibrin and fused strands and has an alternate pattern of thick, non-porous sections, long strands, and bundles with pores. There are also kinked fibres and bundles to be found. It has a very high fibre density on both sides of the membrane when compared to native fibrin clots. PRFM was used as a therapy technique for the first time in furcation type defects, taking into account all of these qualities to reduce the extra expense of the barrier membrane and to prevent bacterial contamination owing to exposure of the membrane. Due to the membrane’s capacity to maintain space, the synergistic impact of membrane and bone graft gave advantages over clinical criteria such as reduction in PD and gain in CAL in the current trial.

Studies stated that horizontal defect depth of 5 mm or more demonstrated less probability of complete closure (52%) and horizontal defects depth around 4 mm or less than presented higher chances of complete furcation defect closure (84%). ^53^ In the present study, presurgical measurement of HPD was from 3-6 mm thus showing lesser chances of complete defect closure.

In the present study, both the test group (n=10; 83.33%) and the control group (n=8; 66.6%), the proportion of defects changed from class II to class I demonstrated extraordinary regeneration capacity. GTR treatment has been shown in literature to aid in the conversion of class II to class I furcation, hence improving the tooth’s long-term prognosis. ^54^ Compared to grafting alone, combined treatment offers several advantages. It holds the graft in place in the defect, supports the membrane in the proper position, and improves epithelial exclusion. In furcation management, long-term studies showed a gain in attachment of up to 4.5 mm with GTR treatment. In comparison to baseline values, all remaining defects in the test group (n=2; 16.66%) and control group (n=4; 33.33%) demonstrated a substantial reduction in defect depth.

To the best of our knowledge, no research has investigated the efficacy of DFDBA and PRFM in FI. Therefore, comparison of this combination approach in furcation defects was made. As a result, it stands for an exogenic comparison of DFDBA and PRFM with a well-established surgical technique for furcation defect regeneration.

## Conclusions

In our study, clinical as well as radiographic evaluations are carried out to evaluate the regenerative potential which limit the assessment. Thus, to govern the stability of the outcomes attained in this study, histological evidence over the long span of time is desirable. Also, larger sample size needed to be planned to quantify the regeneration.

### Registrations number - CTRI/2020/06/025979

Protocol details - JOURNAL OF CRITICAL REVIEWS ISSN - 2394-5125 VOL 6, ISSUE 6, 2019 Comparative Evaluation of Platelet Rich Fibrin Matrix membrane and Collagen membrane with Demineralized Freeze Dried Bone Allograft in Class II Furcation Defects using CBCT – A Randomized Controlled Clinical Trial DR. CHITRIKA SUBHADARSANEE 1, DR. PRASAD V. DHADSE 2.

## Data Availability

Figshare: Master chart thesis.xlsx. https://doi.org/10. 6084/m9.figshare.22153985.v1. ^45^ This project contains the following underlying data:
- Master chart thesis.xlsx (Data taken from both test and control groups) Figshare: Surgical procedure for test group. https://doi.org/10.6084/m9.figshare.22559734. ^43^ This project contains the following extended data:
-
Figure 1-Measurement of VPD using UNC-15 probe. JPG -
Figure 2-Full thickness flap reflection. JPG -
Figure 3-Intrasurgical vertical defect depth measurement. JPG -
Figure 4-DFDBA placed in furcation defect. JPG -
Figure 5-PRFM placed in the furcation defect. JPG -
Figure 6-Flap sutured. JPG Figshare: Surgical procedure for control group. https://doi.org/10.6084/m9.figshare.22629394. ^44^ This project contains the following extended data:
-
Figure 1- Measurement of VPD using UNC-15 probe.jpg -
Figure 2- Full thickness flap reflection.jpg -
Figure 3- Intrasurgical vertical defect depth measurement.jpg -
Figure 4- DFDBA placed in furcation defect.jpg -
Figure 5- GTR membrane placed in the furcation defect.jpg -
Figure 6- Flap sutured.jpg Figshare: Clinical and radiographic evaluation of test group. https://doi.org/10.6084/m9.figshare.22630369. ^47^ This project contains the following extended data:
- Pre operative Vertical defect depth and defect width (test group).png - Pre operative Horizontal Defect Depth (test group).png - Post operative Vertical Defect Depth and Defect Width (test group).png - Post operative Horizontal Defect Depth (test group).png Figshare: Clinical and radiographic evaluation of control group. https://doi.org/10.6084/m9.figshare.22630405. ^48^ This project contains the following extended data:
- Pre operative Vertical defect depth and defect width (Control group).png - Pre operative Horizontal Defect Depth (control group).png - Post operative Vertical Defect Depth and Defect Width (control group).png - Post operative Horizontal Defect Depth (control group).png Figshare: CONSORT checklist. https://doi.org/10.6084/m9.figshare.22300159.v2. ^39^ This project contains the following extended data:
- CONSORT_checklist.docx Figshare: CONSORT Flow Diagram. https://doi.org/10.6084/m9.figshare.22352815. ^40^ This project contains the following extended data:
- CONSORT flow diagram.docx Data are available under the terms of the Creative Commons Attribution 4.0 International license (CC-BY 4.0).
