# Supplementary material for: Comparative evaluation of platelet rich fibrin matrix (PRFM) membrane and collagen membrane with demineralized freeze-dried bone allograft (DFDBA) in the treatment of mandibular class II furcation defects: A randomized controlled trial
Source: F1000Res. 2023 Oct 20;12:1387. [Version 1] doi: 10.12688/f1000research.131974.1 (PMC12419145; doi:10.12688/f1000research.131974.1)
Supplement: Peer review history

# Reviewer report: Reviewer response for version 1

**DOI:** 10.5256/f1000research.144865.r400582

**Published:** 2025-09-04

**Authors:** Maha Sh Mahmood, Hadeel Mazin Akram

**Affiliations:** 1 University of Baghdad, Baghdad, Baghdad Governorate, Iraq; 2 Periodontics, University of Baghdad Bab Al-Moadham Campus College of Dentistry, Baghdad, Baghdad Governorate, Iraq

I have carefully reviewed your manuscript titled “Comparative evaluation of platelet rich fibrin matrix (PRFM) membrane and collagen membrane with demineralized freeze-dried bone allograft (DFDBA) in the treatment of mandibular class II furcation defects: A randomized controlled trial.”

 First, I commend you on conducting a well-designed randomized controlled trial that addresses an important question in periodontal regenerative therapy. The topic is clinically relevant, the methodology is clearly described, and the inclusion of CBCT for radiographic analysis is a particular strength. Overall, this is a fine study that makes a valuable contribution. I would like to offer the following suggestions to strengthen your work further:

**Major Comments**

  1. Conclusions vs. Data

 While both groups showed statistically significant improvements from baseline, there were no significant differences between PRFM and collagen membranes for primary or secondary outcomes. The conclusion should be refined to reflect this equivalence rather than implying clear superiority of PRFM. Highlighting that PRFM is a viable and cost-effective alternative to collagen membrane would be a strong, evidence-based message.

  2. Sample Size & Power

 The study includes only 24 sites. Although the sample size calculation was provided, the trial may be underpowered to detect subtle but clinically relevant differences. Please discuss this limitation more explicitly in the discussion.

  3. Terminology of “Regeneration”

 Since no histological evidence was collected, the use of the term “regeneration” should be cautious. Alternative terms such as “clinical improvement” or “healing” may be more appropriate.

  4. Novelty

 You state that this is the first study evaluating PRFM with DFDBA in furcation defects. Please elaborate on how your study is novel in design, scope, or clinical application to emphasize its contribution.

  5. Methodological Details

 For reproducibility, include company names/manufacturers for the 0.2% chlorhexidine gluconate, periodontal dressing, and amoxicillin. It would also be helpful to cite references for both the classification of “moderate to advanced chronic periodontitis” and for the PRFM preparation protocol.

  6. Presentation of Results

 Your tables contain 3-month follow-up data, but the discussion gives limited attention to these results. A brief commentary on the healing trajectory and what these early findings suggest would add depth. Additionally, reorganizing tables in a more chronological flow may improve readability.

**Minor Comments**

  • There are several grammatical and language issues throughout. A thorough English language edit is recommended.

  • Consider including the following article in your introduction alongside reference No. 16:

 The efficacy of non-surgical platelet-rich fibrin application on clinical periodontal parameters and periostin level in periodontitis: Clinical trial. https://doi.org/10.1111/jcmm.17675

 Summary Recommendation

 Approved with reservations. Your data strongly support that PRFM performs comparably to collagen membrane and can be considered a cost-effective, clinically effective option. Addressing the above points will further improve the clarity, impact, and scientific rigor of your manuscript.

Is the work clearly and accurately presented and does it cite the current literature?

Yes

If applicable, is the statistical analysis and its interpretation appropriate?

Yes

Are all the source data underlying the results available to ensure full reproducibility?

Yes

Is the study design appropriate and is the work technically sound?

Yes

Are the conclusions drawn adequately supported by the results?

Partly

Are sufficient details of methods and analysis provided to allow replication by others?

Yes

Reviewer Expertise:

Periodontics

We confirm that we have read this submission and believe that we have an appropriate level of expertise to confirm that it is of an acceptable scientific standard.

# Reviewer report: Reviewer response for version 1

**DOI:** 10.5256/f1000research.144865.r217529

**Published:** 2024-05-14

**Authors:** Vidya Sagar

**Affiliations:** 1 KAMINENI INSTITUTE OF DENTAL SCIENCES, TELANGANA, India

**Reviewer’s comments**

**Title: Comparative evaluation of platelet rich fibrin matrix (PRFM) membrane and collagen membrane with demineralized freeze-dried bone allograft (DFDBA) in the treatment of mandibular class II furcation defects: A randomized controlled trial**

 Kindly provide reference how the subjects were selected as moderate and advanced chronic periodontitis.

1. Kindly provide reference for furcation classification.
2. Kindly provide CBCT images as it's primary outcome of the study.
3. Kindly mention the company name of 0.2% chlorhexidine gluconate.
4. Vertical furcation defect measurement (VDD) - kindly justify with reference.
5. Membrane will be stabilized - replace will by was.
6. Kindly mention company name of periodontal dressing.
7. Kindly provide reference for PRFM preparation.
8. Kindly mention amoxicillin 500mg company name.
9. Replace CAL by R-CAL and GML by R-GML.
10. The present study didn't interpret the results of all the parameters at 3 month which were shown in the tables. Only few parameters have been explained.
11. Tables are not in chronological order.

Please submit with the above changes.

 The comments to consider for this study:

1. Strengths: The study design, including randomization and a parallel-controlled setup, adds to the validity of the findings. The use of both clinical and radiographic parameters provides a comprehensive evaluation of the treatment outcomes. The study's emphasis on feasibility aspects such as patient acceptance and satisfactory outcomes adds to its clinical relevance.
2. Limitations: The relatively small sample size of 24 subjects could limit the generalizability of the results. While the study highlights the benefits of PRFM membrane combined with DFDBA, it is essential to consider potential confounding factors that might influence the results, such as patient-related factors or variations in the defect characteristics.
3. Clinical Implications: The findings suggest that PRFM membrane combined with DFDBA could be a promising treatment option for managing mandibular Class II furcation defects. However, further research with a larger sample size and longer follow-up is warranted to establish the broader applicability and efficacy of this treatment approach.

Overall, the study contributes valuable insights into the comparative effectiveness of PRFM membrane and collagen membrane with DFDBA in the treatment of mandibular Class II furcation defects, emphasizing the potential benefits of PRFM membrane in this context. Further research and analysis are necessary to confirm and expand upon these findings.

Is the work clearly and accurately presented and does it cite the current literature?

Yes

If applicable, is the statistical analysis and its interpretation appropriate?

Yes

Are all the source data underlying the results available to ensure full reproducibility?

Yes

Is the study design appropriate and is the work technically sound?

Yes

Are the conclusions drawn adequately supported by the results?

Yes

Are sufficient details of methods and analysis provided to allow replication by others?

Yes

Reviewer Expertise:

PERIODONTICS, BONE REGENERATION,

I confirm that I have read this submission and believe that I have an appropriate level of expertise to confirm that it is of an acceptable scientific standard.
